# Biocompatibility of Advanced Manufactured Titanium Implants—A Review

**DOI:** 10.3390/ma7128168

**Published:** 2014-12-19

**Authors:** Alfred T. Sidambe

**Affiliations:** Bioengineering & Health Technologies Group, School of Clinical Dentistry, University of Sheffield, 19 Claremont Crescent, Sheffield S10 2TA, UK; E-Mail: a.t.sidambe@gmail.com; Tel.: +44-114-271-7890; Fax: +44-114-279-7050

**Keywords:** CP-Ti, Ti6Al4V, titanium, biocompatibility, powder metallurgy, metal injection moulding, additive manufacturing, 3-D printing, cytotoxicity, implants

## Abstract

Titanium (Ti) and its alloys may be processed via advanced powder manufacturing routes such as additive layer manufacturing (or 3D printing) or metal injection moulding. This field is receiving increased attention from various manufacturing sectors including the medical devices sector. It is possible that advanced manufacturing techniques could replace the machining or casting of metal alloys in the manufacture of devices because of associated advantages that include design flexibility, reduced processing costs, reduced waste, and the opportunity to more easily manufacture complex or custom-shaped implants. The emerging advanced manufacturing approaches of metal injection moulding and additive layer manufacturing are receiving particular attention from the implant fabrication industry because they could overcome some of the difficulties associated with traditional implant fabrication techniques such as titanium casting. Using advanced manufacturing, it is also possible to produce more complex porous structures with improved mechanical performance, potentially matching the modulus of elasticity of local bone. While the economic and engineering potential of advanced manufacturing for the manufacture of musculo-skeletal implants is therefore clear, the impact on the biocompatibility of the materials has been less investigated. In this review, the capabilities of advanced powder manufacturing routes in producing components that are suitable for biomedical implant applications are assessed with emphasis placed on surface finishes and porous structures. Given that biocompatibility and host bone response are critical determinants of clinical performance, published studies of* in vitro* and* in vivo* research have been considered carefully. The review concludes with a future outlook on advanced Ti production for biomedical implants using powder metallurgy.

## 1. Introduction

Materials used for biomedical applications exist in different forms and they must possess specific properties to fulfil this role [1]. Materials such as metals are commonly used as implants and such metals have to possess properties which will enable them to function inside the human or animal body [2]. Biomaterials, as they are also known, are expected to have biomechanical properties which are comparable to those of autogenous tissues without side effects. The properties which determine whether a material is suitable for biomedical implant applications include biocompatibility, bioadhesion, biofunctionality and corrosion resistance [2]. In order to ensure safety and to have the desired results, implants and other devices intended for biomedical use are regulated by different bodies globally such as the U.S. Food and Drug Administration (FDA) and the International Standards Organisation (ISO) [3]. The main metallic biomaterials are stainless steels, cobalt alloys, titanium and titanium alloys [1]. This review has been limited to the study of titanium and its alloys because this is a metal whose widespread use has been limited by its high cost due to the multi-step Kroll extraction process of the Ti raw material [4]. Advanced powder manufacturing routes such as metal injection moulding (MIM) have emerged as techniques that can minimise the cost of titanium implant production.

## 2. Titanium as Implant Material

Titanium and titanium alloys exhibit a high specific strength [5], which makes titanium an excellent choice for biomedical applications [6]. Furthermore, titanium is considered to be biocompatible because it has a low electrical conductivity which contributes to the electrochemical oxidation of titanium leading to the formation of a thin passive oxide layer [7]. The oxide layer in turn leads to a high resistance to corrosion. This protective passive layer is retained at pH values of the human body [8] due to titanium having an oxide isoelectric point of 5–6 [1]. In aqueous environments Ti and its oxides have low ion-formation tendency and low reactivity with macromolecules [9]. Titanium alloys are used in biomedical implant devices which replace damaged hard tissue. Some examples of Ti uses in biomedical applications are dental and orthopaedic implants, artificial hearts, pacemakers, artificial knee joints, bone plates, cardiac valve prostheses, screws for fracture fixation, artificial hip joints [1] and cornea backplates [10]. Titanium and titanium alloys have therefore been used widely as biomedical implant materials since the early 1970s and the implants have been available as machined and cast components. The alloys that are preferred for the fabrication of titanium implants are commercially pure titanium (CP-Ti) and titanium alloy Ti6Al4V (Ti-64). CP-Ti has a higher resistance to corrosion and is widely regarded as the most biocompatible metal because of a stable and an inert oxide layer which spontaneously forms when its surface is exposed to oxidising media [1]. Almost all commercially available permucosal dental implants are made from CP-Ti as a result of the pioneering research of Brånemark and his co-workers [11].

The CP-Ti and Ti-64 manufactured via the traditional routes (such as strips, sheets, plates, bars, billets, forgings and wires) are specified according to the American Society for Testing and Materials (ASTM) as grades 1 to 5. Grades 1 to 4 are the unalloyed CP-Ti and grade 5 is the alloyed Ti-64. Table 1 summaries the mechanical properties of titanium according to the ASTM standards F67 [12] and F136 [13] for bars, billets and forgings. Grade 2 titanium is the main unalloyed Ti used in dental implant applications. Grade 2 Ti has a minimum yield strength of 275 MPa and this is the equivalent of yield strength in heat-treated austenitic stainless steels. Grade 5 Ti-64 is the most widely used titanium alloy in biomedical implants where high strength is required [1]. As it can be seen form Table 1, CP-Ti has lower strength whilst Ti-64 is an α + β alloy which offers a higher strength [14].

**Table 1:** Selected mechanical requirements properties of titanium bar for implant [1].

Material	Specification	Tensile strength (MPa)	0.2% Proof stress (MPa)	Elongation (%)	Elastic modulus (GPa)
CP-Ti	ASTM F67 Grade 1	240	170	24	103–107
-	ASTM F67 Grade 2	345	275	20	103–107
-	ASTM F67 Grade 3	450	380	18	103–107
-	ASTM F67 Grade 4	550	483	15	103–107
Ti6Al4V	ASTM F136 Grade 5	860	795	10	114–120

MPa = megapascal, GPa = gigapascal.

As much as CP-Ti and Ti-64 have a number of advantages which have already been mentioned above, they do however have some disadvantages as far as implant applications are concerned in that they both have low wear resistance properties. There have also been concerns that the vanadium contained in the Ti-64 alloy is cytotoxic [2] which means that this alloy is limited to certain appropriate applications and devices [13]. CP-Ti and Ti-64 have an elastic modulus which is considered to be high when compared to the bone [2]. These factors therefore limit the application of CP-Ti and Ti-64 because of the mismatch of the elastic (or Young’s) modulus between the implant and the bones [15]. A low elastic modulus is desirable in implants because it helps to avoid stress shielding and the associated bone resorption [16]. As a result, new titanium alloy compositions which are specifically tailored for biomedical applications have been developed. The first generation of these biomedical implant alloys have included Ti-6Al-7Nb and Ti-5Al-2.5Fe, two alloys with properties similar to Ti-64 that were developed to address concerns relating vanadium’s cytotoxicity [2]. Titanium alloys containing Nb, Ta and Zr (β stabilising elements) with a low modulus, high strength, resistance to corrosion and biocompatibility have also been developed for biomedical implant applications.

Another solution to the problems associated with the high titanium elastic modulus has been to use advanced manufacturing processes such as additive layer manufacturing (ALM) to make highly porous titanium structures of CP-Ti and Ti-64. These porous structures can be tailored to have excellent mechanical properties similar to those of human bone and are usually designed to facilitate bone ingrowth [17]. The elastic modulus of titanium is about 114 GPa while that of cancellous and cortical bone range from 0.5 GPa to a maximum of 20 GPa [18]. The mechanical properties and behaviour of titanium implants can be varied via the volume fraction and size distribution of the pore structures [19] and it has been shown that the elastic modulus of porous titanium decreases with increasing pore size under a compressive force [18].

The increased interest in the advanced manufacturing of titanium has led to the subsequent need for regulations and standards and in response the ASTM and the International Organization for Standardization (ISO) have recently published new standards for ALM and MIM titanium. Table 2 shows the chemical composition requirements for MIM and ALM titanium according to ASTM standards F2989, F2885 [20], F2924 [21], F3001 [22] and ISO 22068 [23] standards. Titanium ASTM F2989 “MIM 1” has the highest purity with 0.20 wt % maximum iron content, which is lower than “MIM 2” and “MIM 3” (0.30 wt % maximum). In comparison to the chemical properties of ASTM F67, the chemical requirements are unchanged except for the oxygen in Grade MIM 3 which is set at 0.3 wt % maximum whereas it is 0.35 wt % in ASTM F67. When comparing the alloyed Ti-64 ASTM standards, the maximum oxygen level is set at 0.13 wt % extra-low interstitial (ELI) for the bars, billets and forgings (ASTM F136). In MIM the maximum oxygen level is 0.2 wt % because in processing MIM there is always oxygen pickup during sintering.

**Table 2:** Chemical composition requirements for metal injection moulding (MIM) and additive layer manufacturing (ALM) titanium according to American Society for Testing and Materials (ASTM) standards.

Specification	Chemical composition (wt %)							
	Al	V	Fe	C	N	O	H	Y
ASTM F2989 MIM 1	-	-	<0.2	<0.08	<0.03	<0.18	<0.015	-
ASTM F2989 MIM 2	-	-	<0.3	<0.08	<0.03	<0.25	<0.015	-
ASTM F2989 MIM 3	-	-	<0.3	<0.08	<0.05	<0.30	<0.015	-
ISO 22068 MIM Ti-400	-	-	-	<0.2	<0.1	<0.4	-	-
ASTM F2885 Grade 5	5.5–6.75	3.5–4.5	<0.30	<0.08	<0.05	<0.20	<0.015	<0.005
ISO 22068 MIM-Ti6Al4V-600	5.0–7.0	3.0–5.0	-	<0.2	<0.1	<0.4	-	-
ASTM F2924 ALM Ti6Al4V	5.5–6.5	3.5–4.5	<0.3	<0.08	<0.05	<0.20	<0.015	-
ASTM F3001 ALM Ti6Al4V (ELI)	5.5–6.5	3.5–4.5	<0.25	<0.08	<0.05	<0.13	<0.012	<0.005

As it can be seen from Table 2 there are currently two alloyed Ti grades made by ALM that are in the standards and that can be used for surgical implant applications. The ASTM F2924-14 [21] and F3001-14 [22] standards cover additively manufactured Ti6Al4V components using full-melt powder bed fusion such as electron beam melting and laser melting [22]. Table 3 shows the minimum mechanical properties of Ti manufactured by ALM and MIM according to ASTM F2924-14, ASTM F3001-14, ASTM F2989-11, F2885-11 and ISO 22068.

In comparison to requirements for unalloyed titanium bars, billets and forgings, the tensile strength and yield strength requirements are higher in ASTM F2989 MIM whereas the elongation requirements are lower. This is because in the as-sintered state Ti MIM carries some residual porosity [24]. The tensile strength and yield strength requirements in Ti-64 MIM are lower than in bars, billets and forgings whereas it is the same in Ti-64 ALM. The requirements for elongation are lower with Ti-64 ALM.

**Table 3:** Mechanical properties of CP-Ti MIM, Ti-64 MIM and ALM.

Material	Specification	Tensile strength (MPa)	0.2% Proof stress (MPa)	Elongation (%)	R of A * (%)
MIM CP-Ti	ASTM F2989 MIM 1	370	315	23	25
-	ASTM F2989 MIM 2	420	360	17	20
-	ASTM F2989 MIM 3	495	390	10	15
-	ISO 22068 MIM Ti-400	500	400	5	
MIM Ti6Al4V	ASTM F2885 Grade 5	780	680	10	15
-	ISO 22068 MIM-Ti6Al4V-600	800	600	3	-
ALM Ti6Al4V	ASTM F2924 ALM Ti6Al4V	895	825	10	15
ALM Ti6Al4V	ASTM F3001 ALM Ti6Al4V (ELI)	860	795	8	25

* R of A = Reduction of Area.

## 3. Advanced Powder Processing of Titanium

As it was mentioned earlier, titanium production is hampered by the high cost in traditional manufacturing processes, and poor workability for complex shape production. This has led to numerous investigations of various potentially lower-cost processes [4]. Although there are various powder metallurgy routes of processing titanium as have been listed by Froes [25], this review is confined to the relatively new areas of ALM and titanium MIM.

Advanced manufacturing routes such as ALM and Ti MIM are processes which offer design flexibility and cost-saving respectively, for fabricating products that have complicated shapes with a very high accuracy of size. ALM and MIM are distinct from traditional machining techniques, which mostly rely on the removal of material by methods such as cutting or drilling (subtractive manufacturing) [26]. Thus, ALM and MIM are increasingly being employed as processes for fabricating surgical implants including orthopaedic and dental implants. With increasing demand for medical implantation, there is increasing need for implants that offer reliability, adequate mechanical properties and that offer unique properties (such as patient specific implants) as well as comfort. MIM is a processing route that offers reduction in costs, with the added advantage of near net-shape fabrication as reported by Ferri *et al.* [27] and Ebel* et al.* [28]. On the other hand ALM is used to make patient specific, complex, cellular and functional mesh arrays implants or bone substitutes [29].

The aim of this review is to summarize existing literature and report on the use of advanced manufacturing to fabricate titanium alloy implants. Emphasis is placed on the surface finishes, porous structures and various* in vitro* and* in vivo* biocompatibility studies that have been carried out on the advanced manufactured implants. The development of new processes and materials does not typically include biocompatibility testing until the prototype stage is reached. Regulation agencies such as the Food and Drug Administration (FDA) in the USA require biocompatibility testing per ISO 10993 or ASTM F748 prior to device approval [30]. Therefore there is a need to carry out biocompatibility testing in any material or new material processing method [30].

### 3.1. Additive Layer Manufacturing

**Figure 1:**
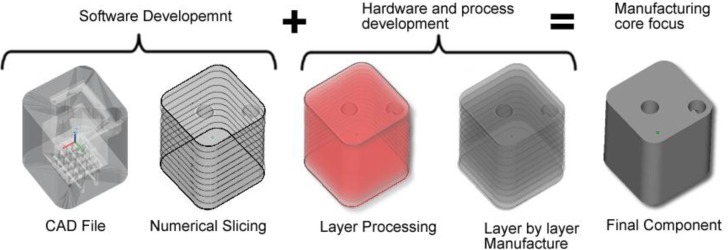
Flow diagram of ALM system from computer-aided design (CAD) file through to component manufacture.

Additive layer manufacturing encompasses a group of technologies that have emerged in the last decade. In ALM, the material is added one cross-sectional layer at a time to create an object [31]. ALM uses the additive method to make a three-dimensional solid object of almost any shape from a computer-aided design (CAD) model. The CAD file model which constitutes the part geometry is created and once optimised, the CAD file is then “sliced numerically” into the layer thickness the machine will build in. After that it is transferred to the ALM machine’s software and loaded to the ALM machine allowing a file based build to begin. Layers of material are laid down successively with each layer corresponding to a different shape. Figure 1 illustrates the flow diagram of ALM system from CAD file through to component manufacture.

ALM technologies include fused deposition modelling, laser micro-sintering, direct metal laser sintering (DMLS), three-dimensional (3-D) laser cladding, electron beam melting (EBM), and electron beam sintering (EBS) [26]. This review looks in detail at the two systems which have the capability to manufacture Ti, namely the EBM and DMLS and briefly at other ALM techniques.

#### 3.1.1. Electron Beam Melting (EBM)

**Figure 2:**
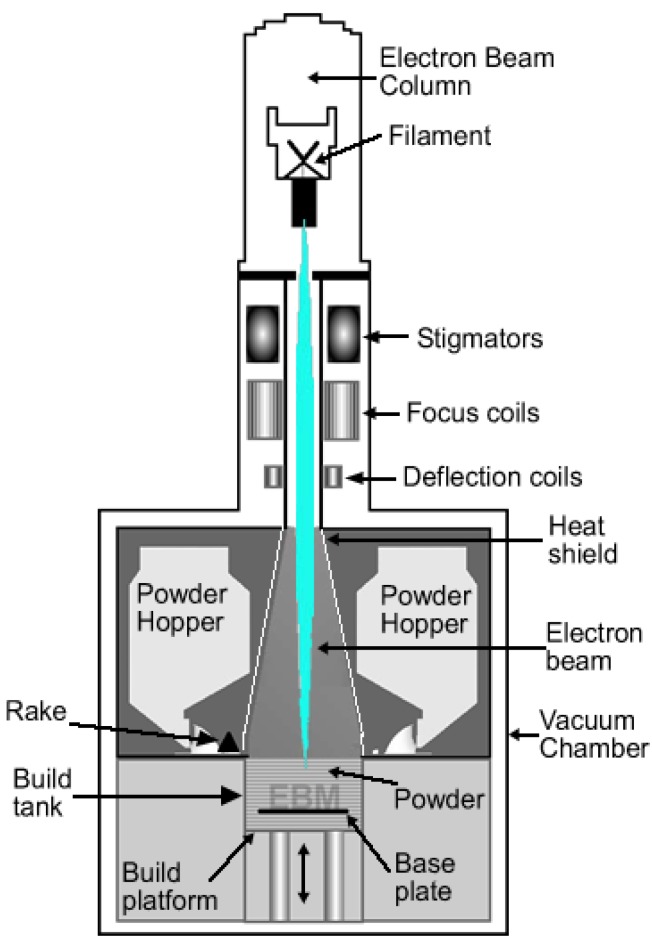
Schematic drawing of an electron beam melting system.

Electron beam melting (EBM) is one of the additive manufacturing techniques mainly used for metallic biomaterials. The EBM system manufactures parts by melting the metal powder layer by layer using a magnetically directed electron beam (up to 3 kW) under a high vacuum atmosphere [32]. It is for this reason that EBM is particularly suited for the manufacturing of titanium and titanium alloy implants. Figure 2 is a schematic drawing of an electron beam melting system.

Part of the challenges of using EBM in the manufacturing of surgical implants is to optimise the surface finish of the final components. This highly depends on the EBM processing parameters such as beam current, part orientation and powder particle size. It is widely known and accepted that the surface topography of biomedical implants affects the biocompatibility because it influences the cell attachment, proliferation, and differentiation. The performance of biomedical implants and their biocompatibility depends very much on the initial interaction between surfaces of the implants and the biological environment [33]. As a result, there are a number of studies that have been carried out to determine the biocompatibility of titanium implants manufactured via EBM.

Ponader* et al.* [33] manufactured Ti-64 components with different surfaces and porosity using EBM and then they assessed,* in vitro*, the surface matrices for cell attachment, proliferation, and differentiation using human fetal osteoblasts (hFOB 1.19). In that study the cell proliferation was found to be more pronounced in the Ti-64 samples which were compact than on the porous samples. These Ti-64 compacts consisted of adherent partly molten titanium particles on the surface, leading to a surface roughness (Ra) of ≤24.9 μm. In porous samples with highly rough surfaces (Ra ≥ 56.9 µm) there was reduced proliferation of the hFOB cells. The authors found that cell differentiation markers were not influenced by the surface roughness. Surface characteristics of titanium could easily be changed by EBM in order to further improve proliferation [33]. This study also concluded that the wear resistance must be optimized and that further investigations were necessary to clarify whether the observed* in vitro* biocompatibility can also be observed* in vivo*, where bone tissue can grow into the EBM structures. The authors then carried out a follow on study where they assessed EBM smooth compacts and porous Ti-64 structures as scaffolds for bone formation,* in vivo* [17]. The implants were placed into defects in the frontal skull of 15 domestic pigs. After surgery, X-Ray photographs were taken to analyse the microscopic structure of the direct contact between bone and implant surfaces in intervals of 14, 30, and 60 days. The results showed that bone ingrowth was increasing with the intervals as follows: around 14% after 14 days, 30% after 30 days and 46% after 60 days in both smooth compact and rough porous implants. There was less bone-implant contact around compact specimens (6%) than around porous specimens (9%) after the 60 days. In the study the authors succeeded in demonstrating the suitability of highly porous titanium implants as scaffolds for bone ingrowth especially in orthopaedic and maxillofacial applications [17].

Whilst Ponader* et al.* [17]* in vivo* study showed relatively low osseointegration within the first 60 days, another* in vivo* study which examined the bone-to-implant contact of EBM Ti-64 implants was carried out by Thomsen* et al.* [34] and this showed the bone-to-implant contact as 29%–41% after 6 weeks in rabbits. After producing EBM Ti-64 implants with an increased surface roughness, the authors machined a selected number of samples and also included conventionally produced bulk Ti-64 material in their study. The results of the early bone response in rabbits showed that the as-EBM Ti-64 implants showed no significant difference in tissue response when compared to the conventional wrought titanium alloy implants and the machined EBM Ti-64. The as-EBM Ti-64 implant specimens had an area surface roughness (*S_a_*) greater than 15 µm. Also revealed in this study were the results of the chemical and mechanical properties of the electron beam-melted Ti-64 material which were within the ASTM F136 specifications. This study was done prior to the issuing of the ASTM 3001 standard.

The biocompatibility of polished, unpolished and porous EBM Ti-64 was also studied by Haslauer* et al.* [29] using* in vitro* cell culture. The authors compared the cellular response in discs which were seeded with 20,000 human adipose-derived adult stem cells (hASCs). The assessments showed that the hASCs were alive on all discs after 8 days, that cellular proliferation on porous EBM Ti-64 discs was increased compared to solid polished and unpolished EBM discs and that the release of the pro-inflammatory cytokines (IL-6 and IL-8) was lower for porous EBM discs than for other discs. Additionally, IL-6 and IL-8 releases at day 7 were lower for porous EBM discs than for other discs. The authors were satisfied that the biocompatibility of the EBM Ti-64 discs was comparable to that of the commercial implant types.

In a study which involved the surface modification of implants via coating, Li* et al.* [35] fabricated porous Ti-64 implants via the EBM process after which they used the biomimetic approach to coat the surfaces. The* in vitro* and* in vivo* biocompatibilities were evaluated for implants with and without biomimetic apatite coating. The results of the* in vitro* biocompatibility of the EBM porous titanium were positive for cell attachment, proliferation and cell morphology. The comparison of implants with and without biomimetic coating showed that cell proliferation in porous EBM Ti-64 implants could match that of coated implants. Similarly, the* in vivo* histological analysis showed a comparable rate of both ingrowth and bone formation between EBM Ti-64 and coated Ti-64 after 12 weeks. The analysis of the mechanical properties showed that the EBM porous Ti-64 had a Young’s modulus that is similar to cortical bone (14.5 to 38.5 GPa).

In a study that included another long-term* in vivo* experiment, Palmquist* et al.* [36] evaluated the osseointegration of porous EBM Ti-64 and solid EBM machined Ti-64 cylindrical and disk-shaped implants. The Ti-64 was implanted in sheep, bilaterally in the femur and subcutaneously in the dorsum. After retrieval 26 weeks later, the results showed that both porous and solid implants were osseointegrated and that high bone–implant contact of 57% was observed throughout the porous implant. However, in discussing the effects of surface topography, the authors suggested that it is not always certain that specific biological effects can be assigned to specific surface property.

The above assertion by Palmquist *et al.* [36] is confirmed by the fact that, although they are interlinked, results of the biocompatibility EBM of titanium alloys listed above are varied. Cell proliferation has been shown to be more pronounced in specimens which are compact and with a lower surface roughness, whereas in another case the authors claim the EBM Ti-64 had the biocompatibility properties which matched reference samples [35] and in another case the authors found more proliferation in rougher specimens compared to the reference samples [29]. Nevertheless, the literature survey has revealed to a certain extent that EBM of titanium can be adopted for the fabrication of custom orthopaedic implants. The process can be used in designing implants with specific surface roughnesses and porosity. The biocompatibility studies have shown how the design flexibility can be correlated with bone ingrowth and cell proliferation, for example. The results open up the possibilities of wider use of EBM titanium to reconstruct specific bone defects.

#### 3.1.2. Direct Metal Laser Sintering (DMLS), Selective Laser Melting/Sintering

Direct metal laser sintering (DMLS) is an additive metal fabrication technology that was developed by EOS who are based in Munich, Germany. DMLS is often also referred to using the terms selective laser sintering (SLS) or selective laser melting (SLM).

**Figure 3:**
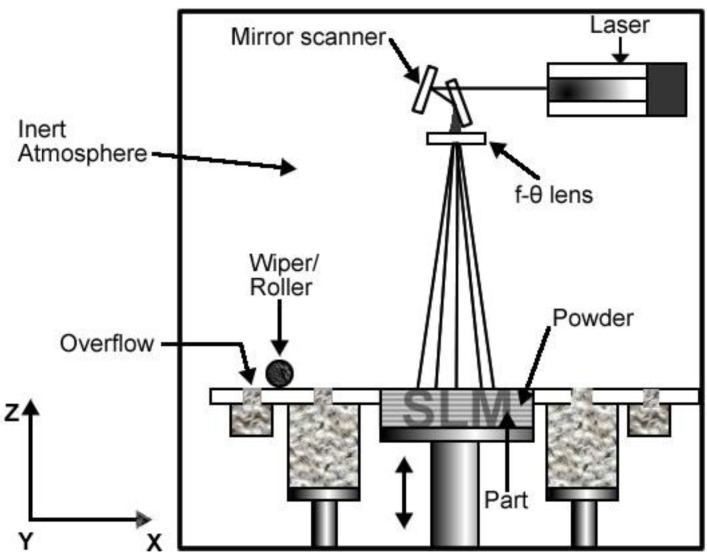
Schematic overview of selective laser melting (SLM) cycle.

In the DMLS system, a high-powered optic laser with a power of 200 W to 400 W is used to fuse metal powder into a solid component based on a 3D CAD file. In a similar manner to the EBM system, components are built from layer to the next layer using the additive method with the layer thickness typically being 20 μm. Figure 3 shows the schematic overview of SLM form of DMLS cycle.

**Figure 4:**
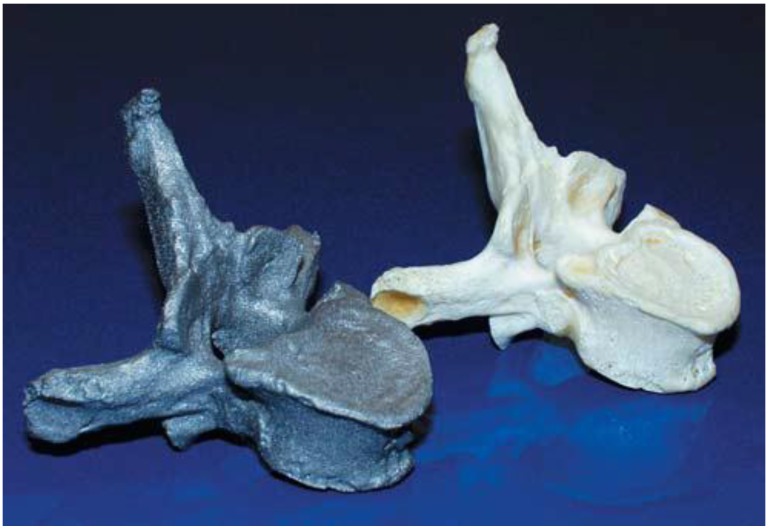
Example of an exact CT-based part of a complex human vertebra processed by SLM using additive manufacturing [37].

The DMLS therefore carries the challenges of manufacturing of biomedical implants with the desired surface finish without the need to post process by polishing* etc.* as on the EBM system. One of the studies that have been carried out in order to investigate the suitability of the titanium DMLS for biomedical implants fabrication was presented by Hollander* et al.* [37]. They presented results which demonstrated the effect of different surface properties on Ti-64 “bone substitutes” made via SLM. In particular, the biocompatibility of SLM Ti-64 material was studied using the primary human osteoblasts cells (HOB). Comparisons were made of the SLM surfaces with the commercial Thermanox^®^ (Nalge Nunc Int., New York, NY, USA) control and conventional bulk titanium. The results showed that the cultured cells attached and proliferated on SLM substrates and the activity of alkaline phosphatase (AP) was also shown to increase after 7 days, but decrease after 14 days [37]. The authors concluded that the increased metabolic activity of osteoblasts on SLM discs compared to the controls may have been due to the greater surface area on the SLM material, which took longer to be covered by the cells. Figure 4 shows the example of an exact computerised tomography (CT) based complex human vertebra processed by the authors using SLM.

Using a DMLS variant called the Selective Laser Sintering (SLS) method, Hollister* et al.* [38] manufactured craniofacial and temporomandibular joint (TMJ) scaffolds for craniofacial reconstruction. The authors developed two interconnected porous architecture designs, one being interconnected spherical pores and the other being interconnected cylindrical pores whose porosity was ranging from 50% to 70%. They used various biomaterials which included an unspecified grade of titanium and presented in the results initial* in vitro* and* in vivo* test data showing substantial bone ingrowth (between 40% and 50% at 6 weeks, between 70% and 80% at 18 weeks) for all scaffolds. The results of the* in vivo* tests showed that the scaffolds, including titanium, were able to support bone regeneration via delivery of BMP-7 transduced human gingival fibroblasts in a mouse model. Furthermore, the titanium scaffolds had mechanical property values lying in between those of cortical and trabecular bone and also supported bone and cartilage regeneration needed to reconstruct craniofacial structures.

Therefore in implantology involving metallic biomaterials like Ti it is important to understand the role of specific surface properties when bone is in contact with an implant. It is for this reason that studies have also been carried out to understand early human bone response to Ti implants, with the more recent studies looking at the advanced manufacturing routes such as DMLS. The early human bone response to a DMLS Ti-64 implant surface has been reported by Mangano* et al.* [39] after they carried out a study where micro-implant was inserted into the anterior mandible of a patient during conventional implant surgery of the jaw as part of the* in vivo* study. After two months of unloaded healing in this study, the micro implant and surrounding tissues were extracted and the structure of tissue was studied by microscopy. The histology showed the peri-implant bone in close contact with the surface of the implant. The mean of bone-to-implant contact was 69.51%. The authors concluded that DMLS Ti-64 is a promising alternative to conventional implant surface topographies [39].

Whilst using* in vitro* studies to demonstrate osseointegration of DMLS titanium, Warnke* et al.* [40] cultured human osteoblasts on SLM-produced Ti-64 mesh scaffolds. Evaluation of the cell occlusion of pores with different widths (0.45 to 1.2 mm) was carried out. The biocompatibility results showed that the osteoblasts had a well-spread morphology and also had multiple contact points. It was found that pore overgrowth increased after 6 weeks of culture where the pore widths were 0.45 and 0.5 mm, and in the course of 3 weeks where the pore widths were 0.55, 0.6, and 0.7 mm. No pore closure was observed on pores of width 0.9–1.2 mm. The authors also found that porosity and maximum compressive load at failure increased and decreased with increasing pore width, respectively. In summary, their scaffolds were biocompatible, and pore width influenced pore overgrowth and resistance to compressive force [40].

The literature survey has revealed to a certain extent that DMLS of titanium can be adopted for the fabrication of custom orthopaedic implants. The results of* in vivo* tests showed that the DMLS scaffolds with unpolished and unmodified surfaces were able to support bone regeneration and that DMLS of titanium as an advanced manufacturing technology is a promising alternative to conventional implant surface topographies. On the other hand the survey has revealed that porous Ti DMLS scaffolds are biocompatible, and that pore width can influence growth around the pores and the resistance to compressive force.

### 3.2. Metal Injection Moulding

Metal injection moulding is a processing route that offers reduction in costs for Ti biomaterials and implants, with the added advantage of near net-shape components. The small to medium sized components with complex geometries that can be manufactured via MIM have the potential to be fully exploited by the implant industry.

**Figure 5:**
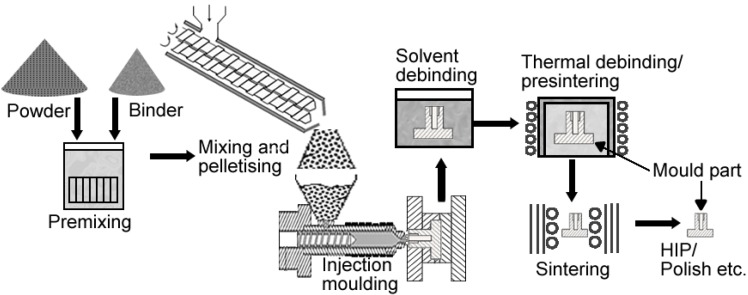
Metal injection moulding flow diagram.

The MIM cycle starts with preparation of a feedstock which consists of mixing fine metallic powder and a binder material. The resulting feedstock is then granulated and an injection moulding machine is used to inject the feedstock into a mould cavity under an elevated temperature (<200 °C) and pressure. Once in the mould the molten feedstock cools and solidifies to produce a green part. The binder is then removed and what is left behind is a highly porous brown part. The brown part is then sintered and shrinks, typically to >95% of the pore-free density (PFD) [24]. More details about MIM have been published elsewhere [41] and the flow diagram for the MIM process is shown in Figure 5.

MIM in manufacturing is well established but the processing of titanium and titanium alloys in this field is still in its infancy because the process has had to depend on high quality starting powders, carbon free binders and improved furnaces for sintering in order to reduce contamination of the titanium. With the necessary improvements, the interest in the manufacturing of titanium for biomedical applications has gathered pace in recent years. Among the challenges in titanium MIM is the production of components with desirable surface finishes, mechanical and chemical properties. In MIM, the quality of the surface finish highly depends on the processing parameters such as mould tool finish and starting powder particle size distribution.

Among the studies that have shown the biocompatibility of implantable MIM Ti is a publication by Sago* et al.* [42]. In the study the authors manufactured Ti-64 devices via MIM and presented the results of the mouldability, the microstructure of the alloy, the mechanical properties as well as the biocompatibility. Their results showed that MIM can produce Ti-64 alloys which meet the specification requirements of implantable ASTM F1472 in the hot isostatically pressed (HIPped) condition. The samples were shown to have passing results for a series of biocompatibility tests, comparable to the wrought grade. Biocompatibility testing for MIM Ti-64 was carried out for hemocompatibility (ISO 10993-4), cytotoxity (ISO 10993-5), sensitization (ISO-10993-10), irritation (ISO 10993-10), systemic toxicity (ISO 10992-11), and implantation (ISO 10993-6) [42].

In a later study, Sago* et al.* [43] also found that the chemical, metallurgical, and mechanical properties of the MIM Ti-64 met the recently published property specifications ASTM F2885 for MIM implantable grades. The samples they manufactured were submitted to a commercial laboratory for biocompatibility testing where they passed a series of biocompatibility tests and were shown to perform at an equivalent level and with no significant differences in biocompatibility when compared with the same grade of implantable wrought alloy.

As more parts are being made from titanium alloys via MIM, the researchers are increasingly producing parts with excellent tensile properties. However, the presence of information containing the fatigue properties which could be useful for biomedical implant applications is scarce. Thus in a study that was intended to manufacture MIM titanium with improved fatigue properties, Ebel* et al.* [28] studied the mechanical, biological, and corrosion properties of specimens manufactured from Ti-6Al-4V-0.5B alloy. There were concerns that there may be unknown reactions in the body environment due to the boron content. Hence corrosion and biocompatibility tests were performed. The human osteosarcoma cell line MG-63 cells were cultured to perform adhesion, proliferation, and viability experiments and the results showed that the alloy Ti-6Al-4V-0.5B satisfies the requirements of a permanent implant material manufactured by MIM [28].

More studies on the suitability of MIM titanium for biomedical implant applications was carried out by Auzene* et al.* [44]. Their study presented results of the surface quality of MIM CP-Ti and MIM Ti-64 and its effect on biocompatibility. Comparisons were carried out between machined grade 4 CP-Ti, MIM grade 4 CP-Ti and MIM grade 4 CP-Ti with three different commercial medical implant coatings (BIOCOAT^®^, BIODIZE^®^ and BIOCER^®^: Steiger Galvanotechnique SA., Chatel-Saint-Denis, Switzerland). Thermanox^®^ was used a control sample. The results revealed that in comparison to machined CP-Ti, MIM CP-Ti had a specific surface roughness which exhibited an excellent biological response. Cell adhesion of the cultured bone explants was poor on the Thermanox^®^ control, and much improved on the MIM-Ti, BIODIZE^®^ and BIOCOAT^®^ but did dramatically increase on BIOCER^®^. The chemical and mechanical properties conformed to ASTM F67 standards for MIM CP-Ti and to ASTM standards F136 and F2885 for MIM Ti-64 [44].

**Figure 6:**
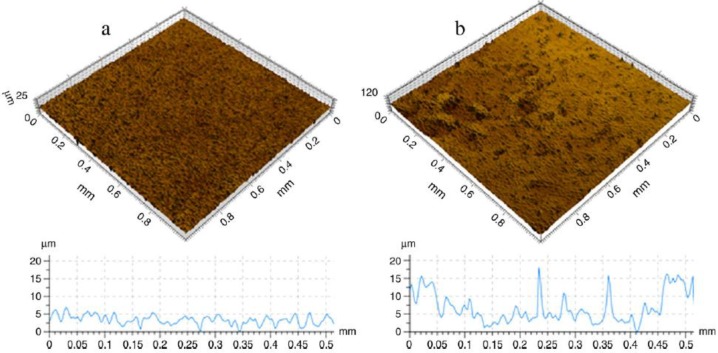
Topographic 3D view of machined CP-Ti (**a**) and MIM CP-Ti (**b**) surfaces, 1 × 1 mm [45].

More results of the study mentioned above were then published by Demangel* et al.* [45]. Their report published results of the biocompatibility of MIM CP-Ti with various anodic oxidation post-treatments as reported by Auzene* et al.* [44]. It was shown that MIM-Ti compared to machined CP-Ti demonstrated a specific surface topography with a higher roughness. MIM-Ti and BIOCER^®^ samples significantly enhanced cell proliferation, cell adhesion and cell differentiation of bone explants compared to CP-Ti. In addition the authors performed some anodisation post-treatment in this study which demonstrated the ability to improve osseointegration through anionic modification treatment. Figure 6 shows the topographic 3D view of machined CP-Ti (a) and MIM CP-Ti (b) surfaces, 1 × 1 mm studied by Demangel *et al.* [45].

**Figure 7:**
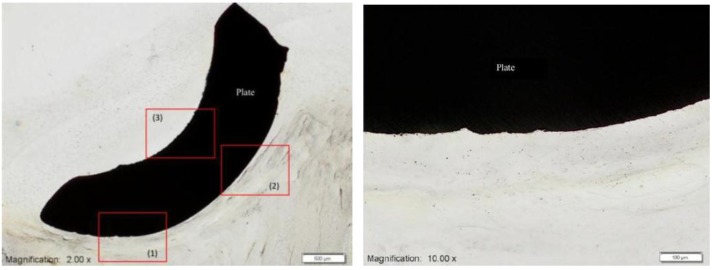
Micrograph showing surrounding tissue on the MIM Ti-64 implant showing continuous contact [46].

Yet more studies relating to the study of the biocompatibility of MIM Ti-64 were carried out by Ibrahim* et al.* [46]. Firstly, the* in vitro* cytotoxicity of MIM Ti-64 was carried out using the mouse fibroblast L929 cell lines. The MIM Ti-64 part was found to be non-toxic to mouse fibroblast cell lines L929. Most of the cells proliferated with numerous filopodia and were attached to the MIM Ti-64 part [46]. Secondly, Ibrahim* et al.* [46] carried out an* in vivo* test of the MIM Ti-64 by placing the implant into the mandible of Macaca fascicularis. Results showed continuous contact between surrounding tissue and the Ti-64 implant as shown in Figure 7 [46].

### 3.3. Other Advanced Manufacturing Techniques

**Figure 8:**
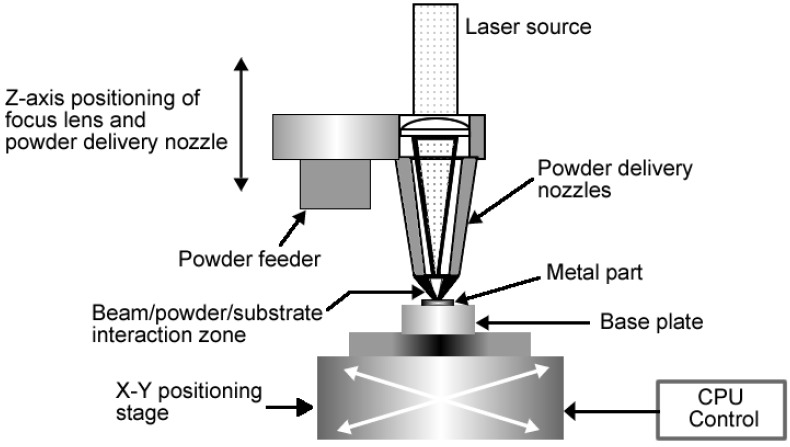
Schematic representation of LENS process.

There are a number of variations of additive manufacturing that have been employed by researchers to make implants. One such technique is Laser engineered net shaping or LENS^TM^ which is a technology developed by Sandia National Laboratories. Similarly to EBM and DSLM, LENS^TM^ is used for fabricating metal parts directly from a computer-aided design (CAD) solid model. The difference is that in LENS^TM^ the metal powder is injected into a molten pool which is created by a focused, high-powered laser beam. Figure 8 is a schematic representation of LENS^TM^ process.

LENS^TM^ produced Ti parts have a rough surface with a macroporous structure and it is this property which allowed for Xue* et al.* [47] to use this method to fabricate porous CP-Ti implants. The effects of the porous structure on bone cell responses were evaluated* in vitro* using human osteoblast cells (OPC1). In the results the cells were found to be well spread on the surface of porous Ti and they formed strong local adhesion with assays showing that the porous CP-Ti surface favours bone cell proliferation. The porous CP-Ti LENS^TM^ implants were also shown to stimulate faster OPC1 cell differentiation compared to a polished CP-Ti sheet. It was stated that this is due to the change in cell morphology within the pores of CP-Ti implant samples. The results also showed that a critical pore size of >200 μm is crucial for cell ingrowth into the pores. When the pore size is <150 μm, cells were found to span directly across the pores [47].

**Figure 9:**
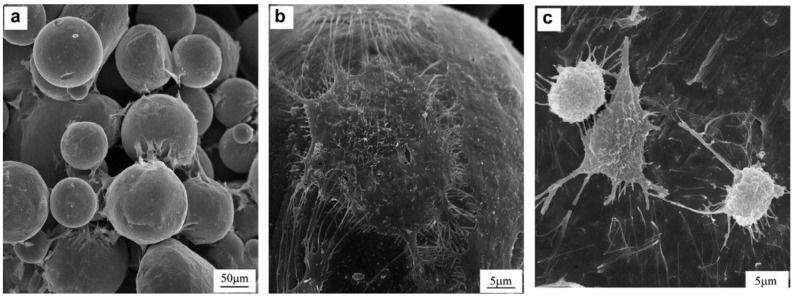
SEM micrographs of OPC1 cells after 3 days of culture on: (**a**,**b**) porous Ti (27% porosity), showing flattened and well-spread morphology; (**c**) Ti plate, showing more rounded shape [47].

This influence of porosity on LENS^TM^ manufactured titanium was also studied by Bandyopadhyay *et al.* [48]. After fabricating porous Ti-64 structures, using the LENS^TM^ technology, the authors sought to demonstrate that advanced manufacturing techniques such as LENS^TM^ can be used to fabricate low-modulus implants, with tailored porosity and which are capable of achieving long-term* in vivo* stability. At a porosity of 23–32 vol %, the corresponding effective elastic modulus was tailored between 7 and 60 GPa, equivalent to human cortical bone. The* in vivo* tests carried out on male Sprague–Dawley rats for 16 weeks revealed that there was significant increase in calcium within the implants, which is an indicator of excellent biological tissue ingrowth through interconnected porosity. Results also showed that the total amount of porosity influences the role in tissue ingrowth [47]. Figure 9 shows SEM micrographs of OPC1 cells and Ti after 3 days of culture on: (a,b) porous Ti (27% porosity), showing flattened and well-spread morphology; (c) Ti plate, showing more rounded shape.

**Figure 10:**
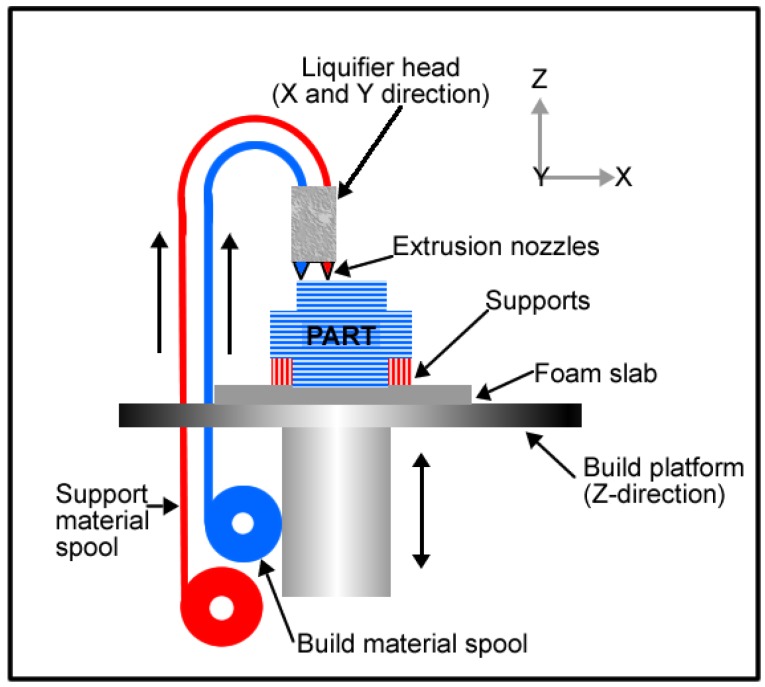
Fused deposition modelling schematic.

In additive manufacturing, there is another technique which is widely used with polymeric materials. This method is known as fused deposition modelling (FDM). In FDM, the material is extruded in building a part layer by layer from a CAD file. The FDM schematic is shown in Figure 10. This technique also gives room for flexibility in design and this is undoubtedly beneficial for implant fabrication because implant size and shape can be tailored leading to the ability to produce patient specific implants.

**Figure 11:**
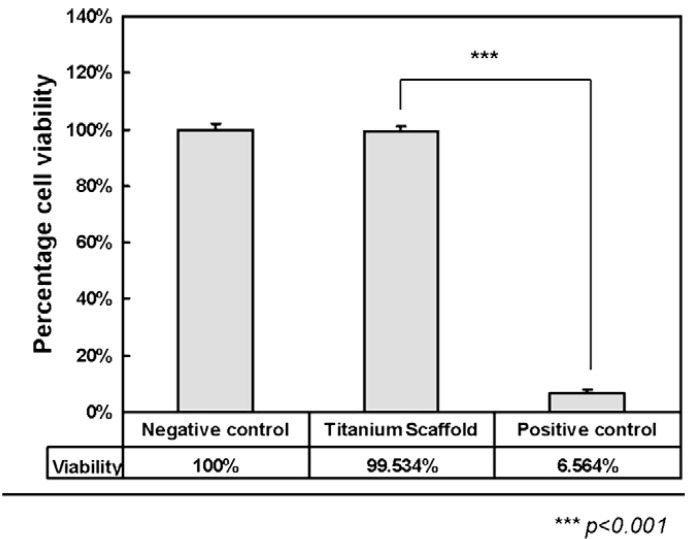
Comparison of cytotoxicity between titanium scaffolds and control cylinder [49].

Wiria* et al.* [49] fabricated titanium implant prototypes using this 3-D printing technique in combination with the debinding and consolidation (sintering) processing that is carried out in MIM. In their study, a 3D Printer normally used for plaster material was used to fabricate green parts of the titanium implant prototype. The CP-Ti powder (325 mesh) was mixed with Poly(vinyl alcohol) (PVA) binder material to make the feedstock. After 3-D printing, the green parts were firstly debound from the PVA binder and then sintered. The subsequent cell culture study which was carried out using the L929 fibroblast cells showed that the porous CP-Ti implant had excellent biocompatibility because of the resulting bone cell attachment and proliferation. The* in vitro* investigation also revealed increased osteogenic differentiation, with little cytotoxicity. Figure 11 shows comparison of cytotoxicity between the CP-Ti scaffolds and control cylinder. The negative control samples used for cytotoxicity tests were in the form of agarose gel cylinders with the same dimension as the titanium implants, whereas phenol samples were used as positive control.

## 4. Conclusions and Future Outlook

Titanium and its alloys have a proven track record as biomedical implants due to their excellent biocompatibility. This is related to the presence of an oxide layer that is typically present on the surface of the material in an oxidising environment. The processing of titanium via the advanced manufacturing technologies of ALM and MIM was until recently inhibited by relatively high costs and the need to demonstrate that the resulting surfaces have the equivalent biocompatibility of implants manufactured using traditional methods. This review has demonstrated that advanced and additive manufacturing can be used successfully to manufacture safe, biocompatible titanium alloy structures for use as medical devices in some applications. This conclusion is supported by a number of* in vitro* and* in vivo* studies. The studies used cultured fibroblasts and osteoblasts in the observation of cell responses to surfaces and also human and animal subjects. Several advanced manufacturing routes have also been shown here to provide an enabling environment for the manufacture of implants with tailored surfaces, porosity and scaffolds. For example, this is a feature that has been used in the fabrication of titanium implants where the elastic modulus matches that of bone tissue. Table 4 and Table 5 summarise the ALM and MIM processing methods and findings. The processing of titanium alloys manufactured via advanced powder manufacturing routes such as additive layer manufacturing (or 3D printing) and metal injection moulding is clearly receiving increased attention and being adopted as alternative to machining and casting. This is as a result of the associated key advantages which include the design flexibility, reduced processing costs, reduced waste, energy efficiency and improved functionality as has been demonstrated in several of the above studies. Therefore, the advanced manufacturing routes collectively represent a significant opportunity to transform the medical device industry. On the other hand, whilst ALM offers design flexibility, the full scale adoption in implant manufacturing may be hindered by the current high cost of machines with metal 3-D printing capabilities and by costs associated with skills training. The field of advanced manufacturing is evolving rapidly, with new technologies and discoveries appearing almost continuously and contributing to a very dynamic field. Furthermore, it has been shown that ALM can produce implants with customised rough surfaces, but in some implant cases such as in joints, a smooth surface is required and ALM and MIM are currently not capable of producing parts with very smooth surface finishes. This means that in some applications, ALM and MIM Ti implants may have to be post-processed or coated to achieve the adequately smooth surfaces. There are also further complexities in that the cell attachment or bone ingrowth, for example, may not be uniform throughout a single Ti implant part.

**Table 4:** Summary of ALM processing methods and findings.

Processing	Alloy	Biocompatibility test	Cell line/implantation	Other comments and references
EBM	Ti6Al4V	*In vitro*	human fetal osteoblasts (hFOB 1.19)	Reduced cell proliferation in highly rough surfaces [33]
EBM	Ti6Al4V	*In vivo*	Frontal skull of domestic pig	More bone contact in more porous samples [17]
EBM	Ti6Al4V	*In vivo*	Rabbit femur and tibia	As-EBM implant response comparable to machined [34]
EBM	Ti6Al4V	*In vitro*	Human adipose-derived adult stem cells (hASC)	Increased proliferation on porous compared to polished and unpolished EBM discs [29]
EBM	Ti6Al4V	*In vitro* and* in vivo*	Osteoblasts extracted from Calvaria of rabbits, Calvaria of rabbits	Proliferation in porous EBM Ti-64 implants matched coated implants [35]
EBM	Ti6Al4V	*In vivo*	Sheep	High bone–implant contact in porous implant [36]
DMLS	Ti6Al4V	*In vitro*	human osteoblasts cells (HOB)	Cultured cells attached and proliferated on SLM substrates [37]
DMLS	Unspecified Ti	*In vitro* and* in vivo*	BMP-7 transduced human gingival Fibroblasts	*In vitro* and* in vivo* test data showing substantial bone ingrowth [38]
DMLS	Ti6Al4V	*In vivo*	Human anterior mandible, minipig mandibular	Peri-implant bone in close contact with the surface of the implant [39]
DMLS	Ti6Al4V	*In vitro*	Human osteoblasts	Osteoblasts well-spread and with multiple contact points [40]
LENS	CP-Ti	*In vitro*	human osteoblast cells (OPC1)	Cells well spread on porous Ti [47]
LENS	Ti6Al4V	*In vivo*	Male Sprague–Dawley rats	Increase in calcium (bone) within implant pores [48]
Modified FDM	CP-Ti	*In vitro*	L929 mouse fibroblast	Excellent bone cell attachment and proliferation [49]

**Table 5:** Summary of MIM processing and findings.

Processing	Alloy	Biocompatibility test	Cell line/implantation	Other comments and references
MIM	Ti6Al4V	*In vitro*	L929 (ISO 10993)	Passing results for ISO10993 tests [42]
MIM	Ti6Al4V0.5B	*In vitro*	MG63 cell	Alloy satisfied requirements of a MIM implant [28]
MIM	CP-Ti and Ti6Al4V	*In vitro*	MC-3T3-E1 pre-osteoblasts	Cell adhesion much improved on the MIM-Ti, BIODIZE^®^ and BIOCOAT^®^ [44]
MIM	CP-Ti	*In vitro*	MC-3T3-E1 pre-osteoblasts	MIM-Ti and BIOCER^®^ had enhanced cell proliferation, adhesion and differentiation [45]
MIM	Ti6Al4V	*In vitro* and* in vivo*	L929 fibroblast and mandible of Macaca fascicularis	Cells proliferated with filopodia and attached to MIM Ti-64 [46]

Published literature clearly demonstrates that advanced powder manufacturing routes produce implants that are suitable for biomedical applications and the field of investigation of the biocompatibility and clinical performance of ALM and MIM metallic implants has been shown in this review to be expanding. However, relative to the biomedical industry and studies, the studies of* in vitro* biocompatibility,* in vivo* tissue response, and clinical performance of advanced manufactured surfaces can be considered to be limited in number. There is also room to improve the scope of these studies because of the continuing innovation in advanced manufacturing technologies. To summarise, the available data is encouraging, even where it is not always based on systematic laboratory or detailed clinical studies. There is little doubt that more research is needed into the biocompatibility and functionality of medical devices manufactured using the full range of additive and advanced manufacturing technologies available today.
